# Editorial: New mechanisms and drugs for the treatment of cardiovascular disease with diabetes

**DOI:** 10.3389/fcvm.2023.1144858

**Published:** 2023-02-20

**Authors:** Shanshan Zhang, Jingwei Li, Yuli Huang, Jie Yu, Xiong-Fei Pan

**Affiliations:** ^1^Section of Epidemiology and Population Health, Ministry of Education Key Laboratory of Birth Defects and Related Diseases of Women and Children, National Medical Products Administration Key Laboratory for Technical Research on Drug Products In Vitro and In Vivo Correlation, West China Second University Hospital, Chengdu, Sichuan, China; ^2^Shuangliu Institute of Women's and Children's Health, Shuangliu Maternal and Child Health Hospital, Chengdu, Sichuan, China; ^3^Department of Epidemiology and Biostatistics, West China School of Public Health and West China Fourth Hospital, Sichuan University, Chengdu, Sichuan, China; ^4^Department of Cardiology, Xinqiao Hospital, Army Military Medical University, Chongqing, China; ^5^Department of Cardiology, Shunde Hospital, Southern Medical University, Foshan, China; ^6^The George Institute for Global Health, Faculty of Medicine, University of New South Wales, Sydney, NSW, Australia

**Keywords:** diabetes, cardiovascular diseases, mechanisms, drugs, cardiovascular therapeutics, oxidative stress, inflammation, SGLT2 inhibitors

Cardiovascular diseases (CVDs) are a leading cause of deaths worldwide (1). Globally, the prevalence of CVDs was estimated to be 608 million cases in 2020. CVDs and diabetes are closely linked: CVDs are major complications for diabetes and share multiple risk factors with diabetes. Concomitant CVDs and diabetes thus should be treated as a substantial subgroup in both clinical treatments and research.

## Risk factors for CVDs and related medical conditions

The presence of uterine fibroids has been reported to associate with hypertension (2). Li B. et al. examined the association between uterine fibroids and CVDs and whether the association was modified by prevalent diabetes using cross-sectional data for 5,509 women from the National Health and Nutrition Examination Survey (NHANES) 1999–2006. Women with uterine fibroids were 1.44 times as likely to have CVDs compared with those without uterine fibroids. Although a statistical interaction was not provided in the article, it seemed that the positive association between uterine fibroids and CVDs was noted only in participants without diabetes but not in those with diabetes. Metabolic syndrome is a constellation of metabolic risk factors for CVDs. Using echocardiography and high-definition impedance cardiography combined with exercise tolerance test, Chen J. et al. found significant structural alteration, apparent overburden of left ventricular work index, and pre- and afterload in non-CVD patients with metabolic syndrome compared with those without metabolic syndrome. Vulnerable plaques (VPs) could contribute to the onset of coronary heart disease (3). It is essential to detect vulnerable plaques early in order to prevent CVDs. Neutrophil-lymphocyte ratio (NLR) is a marker of chronic inflammation, while non-high-density lipoprotein cholesterol (non-HDL-C) is thought to contribute to the formation and development of coronary atherosclerosis. In a cross-sectional analysis among 204 patients with type 2 diabetes, Huang, Yang et al. found that both NLR and non-HDL-C showed independent associations and favorable prediction for coronary artery VPs. If validated in other studies, non-HDL-C and NLR may have the potential to be used as simple predictors for VPs.

Ferroptosis is regulated cell death that is driven by iron-dependent phospholipid peroxidation. Evidence is accumulating that ferroptosis could be involved in multiple diseases. In a review, Guo et al. summarized the current evidence of ferroptosis in the development of CVDs, including cardiomyopathy, atherosclerosis, acute myocardial infarction, myocardial ischemia/reperfusion injury, and heart failure, and assessed the potential underlying mechanisms. In addition, they briefly related their narratives to potential ferroptosis-targeting treatments of CVDs. Pyroptosis is a programmed process that results in non-inflammatory cell death (4), and recent evidence suggests that it has a potential role in diabetic cardiomyopathy (Lu Y. et al.). On this front, Jia et al. reviewed current evidence on pyroptosis mechanisms and pyroptosis-targeting pre-clinical and clinical treatments for diabetic cardiomyopathy.

## Cardiovascular effects of antiglycemic treatments

Since diabetes predisposes to CVDs, whether antiglycemic drugs could be potentially effective for reducing risk of CVDs is still inconclusive (5). Chen C. et al. used real-world data to assess a similar topic in 1,356 patients with diabetes from the NHANES 2017–2020. Oral metformin use was associated with improved cardiovascular health as defined by 5 indicators (smoking, body mass index, physical activity, blood pressure, and total cholesterol). While sodium-glucose co-transporter 2 inhibitor (SGLT2i) has been reported to reduce risks of major adverse cardiovascular events and heart failure hospitalization in patients with type 2 diabetes (6), the potential mechanisms are still elusive. Xi et al. examined cardiovascular effects of 12-week treatment of empagliflozin (a frequently used SGLT2i) in 24 male rats with streptozocin-induced diabetes through a multi-omics approach. Empagliflozin treatment ameliorated lipid accumulation and mitochondrial damage in the myocardium of diabetic rats. In a separate animal study, Zhan et al. examined the mechanism by which dapagliflozin, another SGLT-2i, reduced the risk of atrial fibrillation in diabetes. They found that dapagliflozin alleviated atrial remodeling and reduced the inducibility of atrial fibrillation in rats with streptozocin-induced diabetes, partly through the toll-like receptor 4, interleukin receptor-associated kinase 1, tumor necrosis factor receptor-associated factor 6, and nuclear factor-kappa B inflammatory pathway. For patients with both coronary heart disease and diabetes, glycemic control could be consequential. Education and support platforms may improve compliance to lifestyle improvement and medication use in patients with diabetes. In a parallel-group, open-label, randomized clinical trial among 160 patients with both coronary heart disease and type 2 diabetes, Zhong et al. found that education and support (educational materials and reminders in response to individual blood glucose) through the WeChat group function led to greater reductions in HbA1C, fasting blood glucose, and systolic blood pressure than usual care, which highlights a convenient and low-cost way to improve self-management in coronary heart disease and diabetes.

## Treatments for CVDs complicated by diabetes

Statins are recommended for use in primary and secondary prevention of CVDs in patients with diabetes (7). However, few studies assessed the risk-benefit profiles of statins in patients with concomitant acute myocardial infarction (AMI) and diabetes. In a sample of 1315 patients with AMI and diabetes from the Medical Information Mart Intensive Care-IV (MIMIC-IV), Lu X. et al. showed that statin users had 72% lower in-hospital mortality and 86% lower intensive care unit (ICU) mortality than non-users, and had similar benefits for subgroups with or without hyperlipidemia. In the same study population, Huang, Zhang et al. showed that anti-embolism device therapy was associated with 50% lower 28-day mortality and 52% lower ICU mortality, compared with no such therapy. Left ventricular thrombus (LVT) is a common complication of AMI, and its treatment is still not standardized. Zhu et al. reported successful treatment of splenic infarction and bilateral renal infarction due to multiple peripheral embolization of LVT in a patient with AMI and diabetes using oral anticoagulant combined with percutaneous coronary intervention (PCI). In another case report, Shen, Liao et al. showed that intracoronary artery retrograde thrombolysis (ICART) combined with PCI improved myocardial reperfusion in a patient with ST-segment elevation myocardial infarction (STEMI) and massive thrombus formation complicated with diabetes and hypertension, even if the myocardial infarction exceeded 12 h. In addition, Shen, Wang et al. published a protocol for a randomized controlled trial to investigate whether ICART is more effective than thrombus aspiration or percutaneous transluminal coronary angioplasty in improving myocardial perfusion in 286 patients with STEMI undergoing PCI. The claimed first study of its type could provide evidence for the efficacy and safety of ICART in STEMI patients receiving PCI. The triglyceride-glucose (TyG) index has been reported to be a surrogate biomarker for insulin resistance and be closely related to cardiovascular diseases. In secondary analyses of data from a prospective study among 241 STEMI patients with high thrombus burden, Yu et al. investigated the relationship between the TyG index and post-PCI quantitative flow ratio (QFR), an alternative modality to reflect residual coronary ischemia (8, 9). The authors showed that a high TyG index was an independent risk factor for post-PCI QFR ≤ 0.92 (a cut-off regarded as indicating high risk for adverse cardiovascular events in the work) in STEMI patients (Yu et al.). Although findings from this study were still primitive, it may support the evidence that insulin resistance could be suggestive of residual coronary ischemia in STEMI patients. While the prevalence of dilated cardiomyopathy (DCM) increases, there is still a lack of evidence for the optimal clinical management of recovered DCM (10). Li P. et al. proposed an open-label randomized controlled trial to assess the safety and efficacy of halved vs. original doses of neurohumoral blockades (i.e., “angiotensin converting enzyme inhibitor/angiotensin-receptor blocker/angiotensin receptor-neprilysin inhibitor + beta-blockers”) for patients with recovered DCM. Evidence from this trial could assist decision making for dosages of neurohumoral blockades.

In conclusion, this Research Topic covers a wide variety of articles on etiology, treatments, and prognosis of concomitant CVDs and diabetes. The collective evidence could potentially facilitate clinical research and practice in CVDs complicated by diabetes.

## Author contributions

All authors listed have made a substantial, direct, and intellectual contribution to the work and approved it for publication.
